# Remote versus on-site proctored exam: comparing student results in a cross-sectional study

**DOI:** 10.1186/s12909-021-03068-x

**Published:** 2021-12-20

**Authors:** Vasiliki Andreou, Sanne Peters, Jan Eggermont, Johan Wens, Birgitte Schoenmakers

**Affiliations:** 1 Department of Public Health and Primacy Care, KU Leuven, Academic Center for General Practice, Kapucijnenvoer 7 -Box 7001, 3000 Leuven, Belgium; 2 Evidence Based Practice, EBMPracticeNet, 3000 Leuven, Belgium; 3 School of Health Sciences, Faculty of Medicine, Dentistry and Health Sciences, University of Melbourne, Melbourne, 3800 Australia; 4 Department of Cellular and Molecular Medicine, KU Leuven, 3000 Leuven, Belgium; 5 Center for General Practice/Family Medicine, Department of Primary and Interdisciplinary Care, University of Antwerp, 2610 Wilrijk, Belgium

**Keywords:** General practice, Medical education, Summative evaluation, Online assessment, Remote proctoring

## Abstract

**Background:**

The COVID-19 pandemic has profoundly affected assessment practices in medical education necessitating distancing from the traditional classroom. However, safeguarding academic integrity is of particular importance for high-stakes medical exams. We utilised remote proctoring to administer safely and reliably a proficiency-test for admission to the Advanced Master of General Practice (AMGP). We compared exam results of the remote proctored exam group to those of the on-site proctored exam group.

**Methods:**

A cross-sectional design was adopted with candidates applying for admission to the AMGP. We developed and applied a proctoring software operating on three levels to register suspicious events: recording actions, analysing behaviour, and live supervision. We performed a Mann-Whitney U test to compare exam results from the remote proctored to the on-site proctored group. To get more insight into candidates’ perceptions about proctoring, a post-test questionnaire was administered. An exploratory factor analysis was performed to explore quantitative data, while qualitative data were thematically analysed.

**Results:**

In total, 472 (79%) candidates took the proficiency-test using the proctoring software, while 121 (20%) were on-site with live supervision. The results indicated that the proctoring type does not influence exam results. Out of 472 candidates, 304 filled in the post-test questionnaire. Two factors were extracted from the analysis and identified as candidates’ appreciation of proctoring and as emotional distress because of proctoring. Four themes were identified in the thematic analysis providing more insight on candidates’ emotional well-being.

**Conclusions:**

A comparison of exam results revealed that remote proctoring could be a viable solution for administering high-stakes medical exams. With regards to candidates’ educational experience, remote proctoring was met with mixed feelings. Potential privacy issues and increased test anxiety should be taken into consideration when choosing a proctoring protocol. Future research should explore generalizability of these results utilising other proctoring systems in medical education and in other educational settings.

## Introduction

Maintaining academic integrity in exam settings has been a long-standing challenge for medical educators [1]. In high-stakes medical exams, academic integrity and security is of paramount importance. This type of assessment is suitable for traditional face-to-face education, considering all students are simultaneously assessed, and that discourages cheating. Increased levels of cheating are expected outside the traditional assessment setting. This expectation has deterred offering alternatives for high-stakes medical exams outside the classroom and requiring an on-site proctor.

However, the COVID-19 pandemic has profoundly affected assessment in medical education programs [2]. The COVID-19 countermeasures require sufficient physical distance before and during exams accommodating smaller student groups per exam session. In an online assessment environment, proctoring could be challenging and logistically burdensome. The necessity of guaranteeing safe but academically integral online exams has become imperative more than ever.

Remote proctoring could potentially offer a viable solution for administering high-stakes medical exams. Although it is a common practice in the realm of online courses and formative assessment, it has been underutilised in high-stakes assessments [3]. Literature about remote proctoring in high-stakes exams is relatively diverse. The big body of work focuses on discussing the potential advantages of utilizing remote proctoring for safeguarding academic integrity, test-taker behaviour, and how to combat inappropriate behaviour [2, 4–6]. To a lesser degree, research explores usability and user reactions about remote proctoring and it mostly discusses potential implementation issues, obstacles, and technical difficulties that might arise [7–10]. 

When it comes to evaluation research and exploring the impact of remote proctoring on student outcomes, the available literature mainly compares proctored to unproctored exams [11–14]. This research suggests that unproctored exams are prone to higher levels of cheating in comparison to proctored environments. In medical education, erroneous decisions in high-stakes exams may have harmful consequences to patients and high-quality care. To date, there is little evidence about comparability of exam outcomes between remote and on-site proctored high-stakes exams [15, 16]. Therefore, this study aims at contributing to evaluation research by comparing exam results of an on-site proctored high-stakes medical exam to those of a remote proctored high-stakes medical exam using our proctoring software. Additionally, we report on candidates’ educational experience to further comprehend test-taker behaviour [17–19].

## Methods

### Setting

This study took place within the Flemish Advanced Master’s in General Practice (AMGP), in Belgium.  The AMGP is formally organized and offered by four Flemish universities (KU Leuven, University of Antwerp, University of Ghent, VUB). This collaboration comprises common administration, curriculum, examinations, and residencies, but separate residents’ registration to the university of their choice. Given we have over 900 residents in the AMGP training, exam planning is a complex logistical and administrative process. Therefore, we built more than a decade ago an intelligent, comprehensive, and interactive digital assessment platform. Our platform offers the interface for summative and formative knowledge testing (in six questions formats), Objective Structured Clinical Examination (OSCE)-performance, and proficiency-testing.

Proficiency-testing takes place outside the regular exam regulations and is organised by the four universities together. The test comprises three stages starting with an administrative stage, followed by an actual exam, and finalized by a jury exam for candidates who failed the exam in stage two. The actual exam is an online machine-assisted test that runs on the digital assessment platform and consists of three components: knowledge testing, critical reasoning testing and situational judgment testing. The whole procedure has been running since 2016 and has proven its reliability, validity, acceptability, and feasibility in this format [18, 19].

The necessity of respecting COVID-19 countermeasures but adhering to the original exam format forced us to implement a remote proctored exam to prevent fraud. Therefore, we developed a proctoring software which was tracking and tracing candidates’ behaviour during the exam. The technology we built and applied goes beyond the traditional proctored systems where focus lies on recording sound and image [20].

### Materials and software

**Fig. 1:**
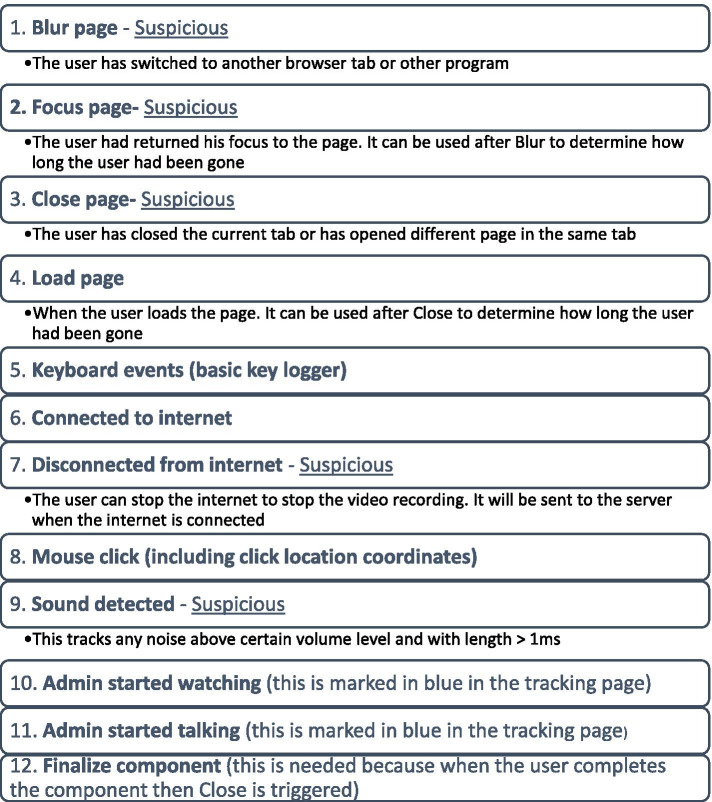
Types of suspicious events tracked and traced by the proctoring software

In collaboration with the developers of the assessment platform and in discussion with the coordinators of the AMGP, we determined the criteria and conditions to design the proctoring software based on our assessment platform. That implies that the software is not immediately available for third parties because of potential compatibility issues. Specifically, we integrated an Application Programming Interface software (Vonage APIs) within our assessment platform. The Vonage APIs software enabled to record and interact with the candidates live. Along with this software, we implemented several metrics to detect events implicating suspicious behaviour (Fig. 1). Suspicious events were defined as: switch to another browser, return to page, close page, disconnection from internet and sound/noise.

During the proficiency-test exam, the system recorded three channels: the computer screen, the camera, and the microphone. The proctoring software operated on three levels: recording of actions, analysis of behaviour and live proctoring. These recordings were immediately encrypted and saved on a secured server. Additionally, the software used an algorithm of pattern recognition in responses, clicking behaviour and timestamp analysis. It also analysed both individual and group behaviour with  correlations across candidates. Every suspicious event was given a score equal to .1. A candidate scoring .5 or higher was considered suspicious. The tracking was stored in the users’ browser and sent to the server after every 20 non-suspicious events or when a suspicious event occurred. When the suspicious rating was above .5, the exam submission was flagged and a report on the suspicious behaviour was downloaded for assessment.

Remote proctoring allowed for optional human oversight during the examination. The human proctor could immediately join the live feed of each candidate to obtain more information or to send a warning via a private message. In case of a software crash, affected candidates were switched to Safe Exam Browser (SEB), which was integrated in our assessment platform. SEB allowed only the exam interface to remain accessible on the machine. To avoid unnecessary pressure on the IT-capacity, we decided not to run the proctoring software along with SEB. In addition to the technical solution, we expected the software to have an impact on candidates’ behaviour regarding fraud prevention. Candidates were comprehensively briefed in advance on how to install the software, on how to test it, and on the specific features of the proctoring software by an animation movie.

A voluntary panel tested the software in two sessions. During the first session, the participants were ordered to behave in a suspicious manner: talking, making noise, turning away from the screen, using the internet, etc. Afterwards, we made the following adjustments to the software and the procedure: we isolated the sound-suspicious level from the other suspicious testing events, because of sensitivity of sound detection. We also increased the overall suspicious level from .5 to 1 to take sound sensitivity into account.

### Data collection

The intervention took place during the regular exam period of the proficiency-test for admission to the AMGP. All participants were candidates applying for admission. To take the exam, candidates were free to register for either remote or on-site participation. On campus, a human proctor was present, and candidates used the campus gear. Candidates that chose a remote option could simultaneously take the exam. For remote proctoring, we engaged an experienced proctoring team of six staff members. The human proctors were able to send online notifications or warnings to candidates who were behaving suspiciously, and they intervened in case of technical issues. The developers of the software were also fully available during the exam to provide technical assistance, if necessary.

The actual exam and the associated procedures were set up as in previous years: all candidates completed the same exam and candidates who failed the machine-assisted exam (the actual exam) were invited to a jury exam one week later. Candidates for whom suspicious behaviour was flagged during the exam or in post-exam analyses were also invited to the jury exam.

**Fig. 2:**
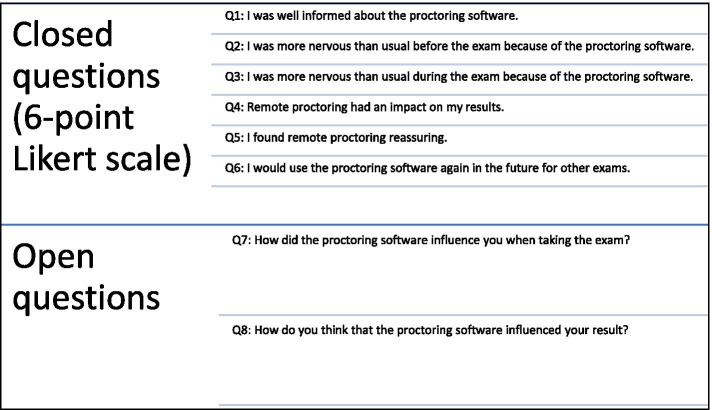
Structure of the questionnaire for exploring candidates’ perceptions about remote proctoring and the software

To get an insight into candidates’ perceptions about proctoring, we administered a post- test survey in the form of an online questionnaire. The questionnaire was sent only to candidates having participated in the remote proctored exam. Filling the questionnaire was on anonymous and voluntary basis. Candidates were asked whether they were taking the proficiency-test for the first time to avoid any effects due to retakes (e.g., test anxiety). Respondents had to specify their level of agreement or disagreement in a 6-point Likert scale for six items regarding proctoring. In addition, candidates could explain how they perceived the influence of proctoring on their exam experience and exam outcome. Figure 2 displays a list of the survey questions. All methods were carried out in accordance with relevant guidelines and regulations. Ethical approval was granted by the Social and Societal Ethics Committee of the KU Leuven with the following approval number: *G-2020-2262-R2(MAR****)***.

### Analysis

We compared exam results based on the proctoring type by using the Mann-Whitney U test, since our data were not normally distributed. The effect size was calculated based on the z-score from the test-statistic, as follows: $=\frac{z}{\sqrt{N}}$ [21]. To analyse the post-test questionnaire, we performed an exploratory factor analysis to understand patterns related to candidates’ perceptions about proctoring [22]. We used an oblique rotation, since we expected some correlation among factors [23]. The reliability of the scale was calculated based on Cronbach’s alpha [24]. We analysed quantitative data with SPSS 27 (IBM SPSS Statistics 27). Qualitative data were thematically analysed by two researchers separately (VA and BS) [25]. Discrepancies in coding were discussed until consensus was reached. Data from open-ended questions were analysed in the software program QSR International’s NVIVO (Release 1.0).

## Results

A total of 593 candidates subscribed to the exam in the four Flemish universities. Four hundred seventy-two (79%) candidates used the proctoring software for a remote exam, while 121 (20%) were present on campus (Table 1). Most candidates registered at the KU Leuven (227) and at the University of Ghent (203) chose to take the exam remotely. The results of both (remote and on-site) groups were comparable (Table 2). Exam results of the remote proctored group (Md = 77.74%) did not significantly differ from those of the on-site proctored group (Md = 78.06%), U = 29,407, z = 0.506, *p* = 0.613, r = 0.02.

**Table 1:** Participation and exam results of remote versus on-site proctored group

	Remote n (%)	On-site n (%)	TT-test pooled
Total number of candidates (*n* = 593)	472 (79,6%)	121 (20,4%)	
Number of candidates per university			
- Leuven	227 (84,1%)	43 (15,9%)	
- Antwerp	29 (35, 8%)	52 (64,2%)	
- Brussels	13 (50%)	13 (50%)	
- Gent	203 (94%)	13 (6%°	
Average exam result	72/100	72,8/100	*P* > 0,15

**Table 2:** Descriptive statistics of the exam results based on the type of proctoring

	k	x (%)	s	Md (%)
Remote proctored exam	100	77.16%	15.24	77.74%
On-site proctored exam	100	76.96%	17.97	78.06%

k = number of exam items; x = mean of exam percentage scores; s = standard deviation; Md = median of exam percentage scores

Overall, we registered and solved 15 technical issues in the remote context (Table 3). Eight of these issues concerned software problems (in particular loading a reading text in a new tab). Two candidates experienced a negative impact on the exam performance due to technical issues. The developers team switched one candidate to SEB mode to complete the exam. Based upon the post exam analyses and after deliberation, the exam coordinator exempted the other candidate of the jury exam.

**Table 3:** Comparison of exam procedure and outcome remote versus on-site

	Remote	On-site
Technical issues	15	1
- with impact on exam	2	0
Type of issue		
- Internet failure	2	0
- Hardware issue	4	1
- Camera crash	1	0
- Software issue	8	0
Average suspicious score	0.4	NA
Median suspicious score	0.3	NA
Number of suspicious candidates		
- Detected by the software	22 (4%)	NA
- Flagged by human proctors	2 (0,04%)	NA
- With non-critical events < 1	455 (96%)	NA
- Without events	15 (3%)	NA
- With noise event	472 (100%)	NA
Interventions during exam		
- Technical intervention	8	1
- Warning to candidate	2 individuals 1 group (background noise)	0 0

In total, the software detected 22 (4%) candidates with a suspicious level > 1. All cases concerned one or more noise-event (background noise). All other non-critical suspicious events consisted of leaving the webpage, closing a page or typing text. Live proctoring and a post exam review of records revealed that all these events occurred hazardously but without fraud purpose. The human proctors flagged two candidates who were typing more than expected (in a multiple-choice exam). After revision of the records, these candidates were using ‘control find’ to search for words in the reading text. During the exam, the proctors intervened eight times for a technical issue, they warned two candidates to stop talking to themselves and they sent a group message to ask to turn down background noise.

**Fig. 3:**
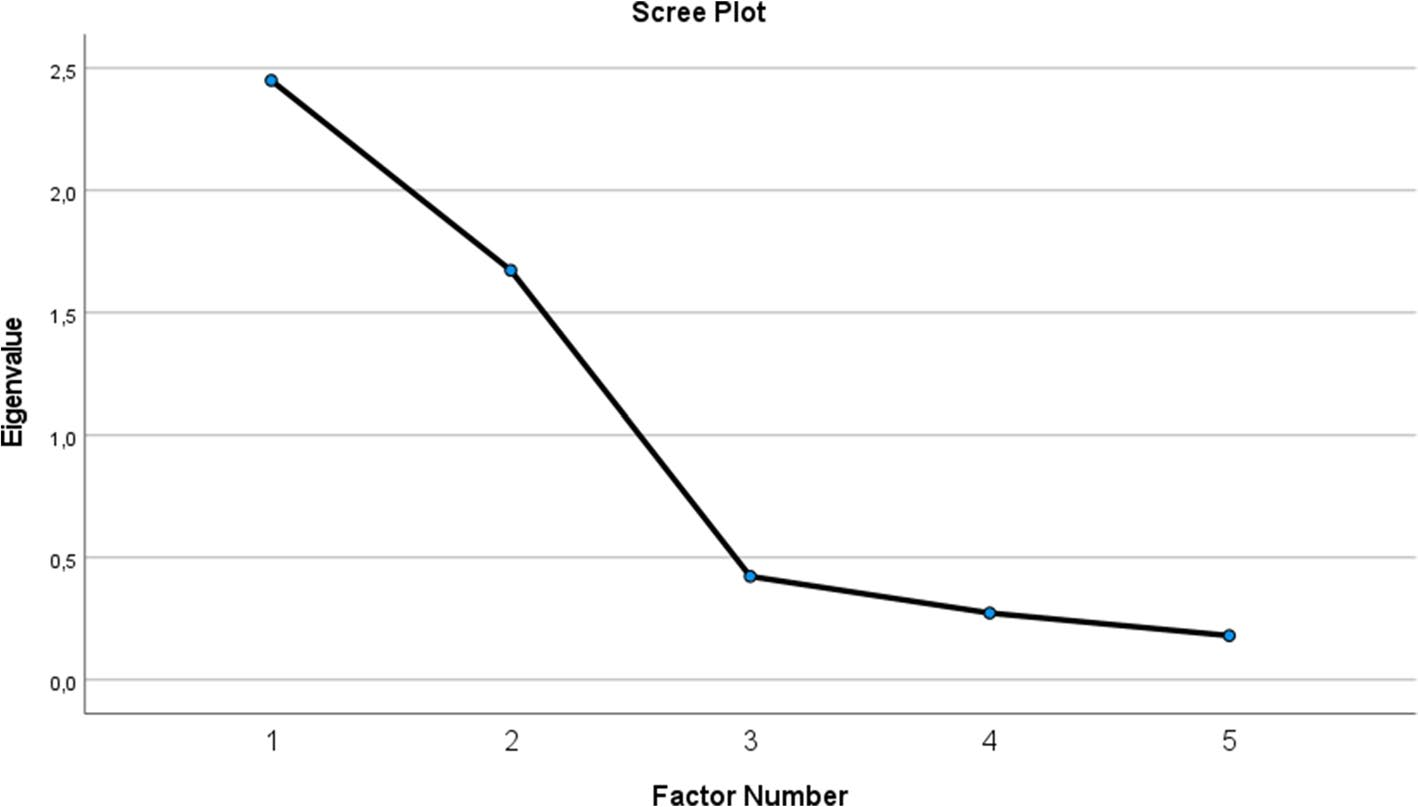
Scree plot of the eigenvalues of the factors

Out of the 472 candidates that used the proctoring software, 304 filled in the post-test questionnaire, 213 women and 91 men. All of them were taking the proficiency test for the first time. An explanatory factor analysis was initially conducted on the 6 items of the questionnaire with oblique rotation. However, one item “I was well informed about the proctoring software” had to be omitted because of a communality lower than .40 [23]. The other five items (communality> .40) were included in a secondary analysis. This analysis yielded a Kaiser-Meyer-Olkin (KMO) of .63 verifying sampling adequacy (Table 4) [22]. Sampling adequacy was also guaranteed by applying the 10:1 rule of thumb subject to item ratio [23]. After analysing the data to obtain eigenvalues for each factor, two factors had eigenvalues over Kaiser’s criterion of 1 explaining 82.46% of the variance. The scree plot also justified retaining two factors (Fig. 3). Table 5 shows the factor loadings after rotation. The items that cluster on the same factors suggest that factor 1 represents candidates’ appreciation of the proctoring software, while factor 2 represents emotional distress because of the proctoring software. The reliability of the questionnaire was calculated based on Cronbach’s alpha as .72.

**Table 4:** KMO and Barlett’s test

KMO and Barlett’s Test	
Kaiser-Meyer-Olkin measure of sampling adequacy	0.632
Barlett’s Test of sphericity	
Approx. chi-square	667,418
df	10
Sig.	.000

**Table 5:** Summary of exploratory factor analysis results from the proctoring software questionnaire (*n* = 304)

Rotated Factor Loadings		
Item	Appreciation of the supervisor app	Emotional distress because of the supervisor app
*Q2: I was more nervous than usual before the exam because of the proctoring software.*	−.017	**.851**
*Q3: I was more nervous than usual during the exam because of the proctoring software.*	−.116	**.963**
*Q4: The proctoring software had an impact on my results.*	.121	**.692**
*Q5: I found the proctoring software reassuring.*	**.973**	.035
*Q6: I would use the proctoring software again in the future for other exams.*	**.738**	−.023
*Eigenvalue*	*2.450*	*1.673*
*% of Total Variance*	*49%*	*33.46%*

Note: Factor loadings over 0.40 appear in bold

From the qualitative analysis, four different themes were discerned. All themes were related to candidates’ emotional well-being. The first two themes referred to stress and anxiety before and during the exam. Some candidates felt more anxious because they feared potential technical problems or running out of time. The third theme was anxiety and stress because of the proctoring software. Candidates mentioned that they felt stress because the software might detect something as fraud, without the intention of fraud. Candidates also admitted that they felt awkward, observed, and distracted knowing that human proctors were monitoring the exam procedure.

Instead of just thinking about a question and the possible answers, I had to constantly remind myself about my behaviour (not to look upwards or left and right). The feeling of constantly being watched was not helpful (Anonymous candidate).

The fourth theme was connected to positive emotions about the proctoring software. Candidates thought that the software was reassuring and that, in case of need, someone could intervene and help.

The software worked pretty well for me, it was rather reassuring that it would be taken into account, if technical problems arose (Anonymous candidate).

Finally, candidates identified some technical issues before and during taking the exam. Before the exam, the main problem was slow internet connection. During the exam, candidates experienced problems, when opening multiple tabs at the same time.

## Discussion

This pilot study aims at comparing two proctoring types, namely remote versus on-site proctoring, for high-stakes medical exams, as to examine potential differences in exam results depending on the exam administration conditions. Overall, the study demonstrates that the proctoring type does not influence exam results.

The results indicate that exam results are equivalent and comparable between the remote and on-site proctored groups. Of great importance in the realm of high-stakes assessment is the fact that the proctoring type did not influence exam outcomes when compared to the on-site proctored group. Remote proctoring could allow for diverse and more flexible ways of administering exams without sacrificing academic integrity and exam quality. Other authors compared scores from proctored exams to scores from traditional exams and have also found no difference in exam outcomes [15, 16].

From an institution’s perspective, there are several logistic benefits for using remote proctoring as suggested by our results. Besides continuation of curricular assessment activities during potential lockdowns, remote proctoring allows assessing many students simultaneously for high-stakes exams, while maintaining academic integrity. This could likely result in reducing costs and decreasing bureaucratic administration. Nevertheless, potential technical issues that might arise demand a well-trained supporting team. It should be recognised that this supportive evidence relates to a sophisticated and complex proctoring software and to real-time human proctors. Therefore, different proctoring scenarios might yield different results.

Regarding candidates’ educational experience, the findings from the exploratory factor analysis indicate that remote proctoring was met with mixed reactions. Considering that candidates took the exam during the pandemic, using a proctoring software seems to have provided reassurance that communication with the university was established, in case of technical difficulties. However, candidates also experienced emotional distress because of proctoring. Similar results have been indicated in previous research about students’ perceptions on remote proctoring [26].

The thematic analysis of the qualitative data provided a more in-depth insight of candidates’ perceptions, especially on emotional wellbeing and test anxiety. A potential explanation for this is that candidates were not sure about what the software could detect as fraud and suspicious behaviour. Hence, more clear instructions could be beneficial for reducing stress when administering remote proctored exams. Consistent with other studies on educational experience of remote proctoring, candidates’ responses also raise issues of privacy, as the feeling of being observed and recorded was debilitating [26–28]. Nevertheless, some candidates experienced proctoring positively stating that they would consider using the software for future exams as well. The direct connection with the university through the software seemed to reassure and comfort candidates that assistance was available, in case of technical issues.

Lastly, the first and second theme relate more to exam anxiety, and specifically to the format of the exam rather than taking the exam remotely. Therefore, they fall outside the scope of this paper, and are not extensively discussed. Regarding technical issues, candidates’ comments showed that software issues were the most frequent, confirming what human proctors observed during the exam.

### Limitations

A limitation of this study is that we cannot rule out the risk of bias stemming from a non- randomized intervention. However, to be in accordance with exam regulations, participants had to choose whether they wanted to take their exams remotely or not. Also, we may assume that this risk is low, since exam results were comparable between the two groups. From other authors we also know that candidates’ preference for computerized or paper-based exams does not influence exam outcomes [29]. Another limitation of our study is the low number of questions included in the questionnaire. Although the questionnaire could be considered reliable based on the Cronbach’s alpha calculation, repeated measurements are necessary to confirm reliability of the scale.

## Conclusion

The COVID-19 pandemic rendered the necessity of utilizing remote proctoring for administering medical exams as an imperative. A comparison of exam results between remote and on-site proctoring indicates that remote proctoring could be a viable solution for administering high-stakes medical exams. A sophisticated proctoring software registering behaviour and recording sound and image to prevent fraud has proven to be efficient without affecting exam outcomes. Admittedly, potential privacy issues and increased test anxiety influencing educational experience should be considered when determining and choosing remote types of proctoring. Future research should explore generalizability of these findings by applying and examining different proctoring systems in medical education and in different educational settings.

## Data Availability

The datasets used and/or analyzed during the current study are available from the corresponding author on reasonable request.

## Abbreviations

AMGP: Advanced Master’s in General Practice
VUB: Vrije Universiteit Brussel
OSCE: Objective Structured Clinical Examination
GP: General Practitioner
API: Application Programming Interface
SEB: Safe Exam BrowserMd = median of exam percentage scores
KMO: Kaiser-Meyer-Olkin

## References

[1] Simpson E, Yu K. Closer to the truth: electronic records of academic dishonesty in an actual classroom setting. Ethics & Behavior - ETHICS BEHAV. 2012;22.

[2] Hamamoto Filho PT, Bicudo AM, Cecilio-Fernandes D. Preserving Cornerstones of Student's Assessment in Medical Education During COVID-19. Front Psychol. 2021;12:591152-. 10.3389/fpsyg.2021.591152 PMC8062750 33897520

[3] (NCCA) NCfCA. Report on the NCCA Assessment of Live Remote Proctoring. Washington, DC 20006: Institute for Credentialing Exellence; 2021.

[4] Berkey D, Halfond J. Cheating, student authentication and proctoring in online programs. 2015, Jul 20 [Available from: https://nebhe.org/journal/cheating-student-authentication-and-proctoring-in-online-programs/.

[5] Dunn T.P. MMFMJ. The remote proctor: An innovatie technological solution for online course integrity. The International Journal of Technology, Knowledge, and Society. 2010;1:1–7.

[6] Langenfeld T (2020). Internet-based proctored assessment: security and fairness issues. Educ Meas Issues Pract.

[7] Gudiño Paredes S, Jasso Peña FdJ, de La Fuente Alcazar JM. Remote proctored exams: integrity assurance in online education? Distance Education 2021;42(2):200–218.

[8] Schoenmakers B, Wens J (2021). Efficiency, usability, and outcomes of proctored next-level exams for proficiency testing in primary care education: observational study. JMIR Form Res.

[9] Lilley M, Meere J, Barker T. Remote live invigilation: a pilot study. J Interact Media Educ. 2016;2016.

[10] Castaño M, Noeller C, Sharma R. Implementing remotely proctored testing in nursing education. Teach Learn Nurs. 2020.

[11] Hollister KK, Berenson ML (2009). Proctored versus unproctored online exams: studying the impact of exam environment on student performance. Decis Sci J Innov Educ.

[12] Daffin LWJ, Jones AA (2018). Comparing student performance on proctored and non-proctored exams in online psychology courses. Online Learning Journal.

[13] Wright NA, Meade AW, Gutierrez SL (2014). Using invariance to examine cheating in unproctored ability tests. Int J Sel Assess.

[14] Dendir S, Maxwell RS (2020). Cheating in online courses: evidence from online proctoring. Computers in Human Behavior Reports.

[15] Weiner JA, Hurtz G, editors. A Comparative Study of Online Remote Proctored Versus Onsite Proctored High-stakes Exams2017.

[16] Sam AH, Reid MD, Amin A (2020). High-stakes, remote-access, open-book examinations. Med Educ.

[17] Ogilvie RW, Trusk TC, Blue AV (1999). Students' attitudes towards computer testing in a basic science course. Med Educ.

[18] Andreou V, Eggermont J, Gielis G (2020). Proficiency testing for identifying underperforming students before postgraduate education: a longitudinal study. BMC Med Educ.

[19] Schoenmakers B, Wens J (2018). Proficiency testing for admission to the postgraduate family medicine education. J Family Med Prim Care.

[20] Munshi F, Alsughayyer A, Alhaidar S, et al. An online clinical exam for fellowship certification during COVID-19 pandemic. Med Educ. 2020. 10.1111/medu.14267 PMC7301032 32501565

[21] Rosenthal R. Meta-analytic procedures for social research. Thousand Oaks, California1991. Available from: https://methods.sagepub.com/book/meta-analytic-procedures-for-social-research.

[22] Field A (2009). Discovering statistics using SPSS: SAGE publications.

[23] Costello AB, Osborne J (2005). Best practices in exploratory factor analysis: four recommendations for getting the Most from your analysis. Pract Assess Res Eval.

[24] Tavakol M, Dennick R (2011). Making sense of Cronbach's alpha. Int J Med Educ.

[25] Clarke V, Braun V (2016). Thematic analysis. J Posit Psychol.

[26] Milone AS, Cortese AM, Balestrieri RL (2017). The impact of proctored online exams on the educational experience. Curr Pharmy Teach Learn.

[27] Karim MN, Kaminsky SE, Behrend TS (2014). Cheating, reactions, and performance in remotely proctored testing: an exploratory experimental study. J Bus Psychol.

[28] Kharbat FF, Abu Daabes AS. E-proctored exams during the COVID-19 pandemic: a close understanding. Educ Inf Technol. 2021. 10.1007/s10639-021-10458-7 PMC7884061 33613081

[29] Hochlehnert A, Brass K, Moeltner A, et al. Does medical students' preference of test format (computer-based vs. paper-based) have an influence on performance? BMC Med Educ. 2011;11:89. 10.1186/1472-6920-11-89 PMC3213144 22026970

